# Neuropilins define distinct populations of neural crest cells

**DOI:** 10.1186/1749-8104-9-24

**Published:** 2014-11-03

**Authors:** Rachael Lumb, Sophie Wiszniak, Samuela Kabbara, Michaela Scherer, Natasha Harvey, Quenten Schwarz

**Affiliations:** Centre for Cancer Biology, University of South Australia and SA Pathology, Frome Road, Adelaide, 5000 Australia; Medical School, University of Adelaide, Frome Road, Adelaide, 5000 Australia

**Keywords:** Neural crest cell, Neuropilin, Sensory neuron, Sympathetic neuron

## Abstract

**Background:**

Neural crest cells (NCCs) are a transient embryonic cell type that give rise to a wide spectrum of derivatives, including neurons and glia of the sensory and autonomic nervous system, melanocytes and connective tissues in the head. Lineage-tracing and functional studies have shown that trunk NCCs migrate along two distinct paths that correlate with different developmental fates. Thus, NCCs migrating ventrally through the anterior somite form sympathetic and sensory ganglia, whereas NCCs migrating dorsolaterally form melanocytes. Although the mechanisms promoting migration along the dorsolateral path are well defined, the molecules providing positional identity to sympathetic and sensory-fated NCCs that migrate along the same ventral path are ill defined. Neuropilins (Nrp1 and Nrp2) are transmembrane glycoproteins that are essential for NCC migration. *Nrp1* and *Nrp2* knockout mice have disparate phenotypes, suggesting that these receptors may play a role in sorting NCCs biased towards sensory and sympathetic fates to appropriate locations.

**Results:**

Here we have combined *in situ* hybridisation, immunohistochemistry and lineage-tracing analyses to demonstrate that neuropilins are expressed in a non-overlapping pattern within NCCs. Whereas Nrp1 is expressed in NCCs emigrating from hindbrain rhombomere 4 (r4) and within trunk NCCs giving rise to sympathetic and sensory ganglia, Nrp2 is preferentially expressed in NCCs emigrating from r2 and in trunk NCCs giving rise to sensory ganglia. By generating a tamoxifen-inducible lineage-tracing system, we further demonstrate that Nrp2-expressing NCCs specifically populate sensory ganglia including the trigeminal ganglia (V) in the head and the dorsal root ganglia in the trunk.

**Conclusions:**

Taken together, our results demonstrate that Nrp1 and Nrp2 are expressed in different populations of NCCs, and that Nrp2-expressing NCCs are strongly biased towards a sensory fate. In the trunk, Nrp2-expressing NCCs specifically give rise to sensory ganglia, whereas Nrp1-expressing NCCs likely give rise to both sensory and sympathetic ganglia. Our findings therefore suggest that neuropilins play an essential role in coordinating NCC migration with fate specification.

## Background

Neural crest cells (NCCs) are transient embryonic cells that generate a wide spectrum of derivatives. Based on their origin along the anteroposterior axis, NCCs are broadly divided into different populations that give rise to a restricted set of derivatives. Cranial NCCs arise anterior to the fifth somite, and form derivates such as sensory, sympathetic, and parasympathetic ganglia, connective tissue, bone, cartilage, and muscle tendons of the head [1]. Trunk NCCs arise posterior to the fourth somite, and give rise to cell types such as sympathetic and sensory neurons, adrenal chromaffin cells, and melanocytes [2]. Vagal NCCs arise from the level of somites 1 to 7, and produce enteric NCCs that form the neurons and glia of the enteric nervous system [3], and other cell types such as cardiac NCCs (level of somites 1 to 3) that form vascular smooth muscle lining the great arteries and contribute to the aortic–pulmonary septum [4].

Even within these anatomically defined domains, NCCs can be further subdivided by additional properties, including their migration path and their developmental fate [5–7]. For example, cranial NCCs migrate in a segmented pattern to populate specific regions of the face and neck. This segmentation is critical for the correct formation and positioning of cranial NCC derivatives, and is under the control of cell intrinsic guidance receptors and signals provided by the environment in which NCCs travel and ultimately differentiate [8, 9]. Trunk NCCs also migrate along distinct paths that correlate with their developmental fate [5]. Thus, NCCs migrate ventrally, and either stop within the sclerotome to give rise to sensory neurons and glia within the dorsal root ganglia (DRG), or migrate towards the dorsal aorta to give rise to the neurons and glia of the sympathetic nervous system. A proportion of these ventrally migrating NCCs that initially migrate along axonal pathways also give rise to melanocytes [10]. By contrast, NCCs migrating dorsolaterally between the dermomyotome and epidermis give rise only to melanocytes. A fundamental question for this field is how NCCs navigate their environment to position themselves in appropriate locations.

There is mounting evidence from chick and mouse studies supporting the notion that NCCs migrating dorsolaterally are specified at the time of delamination [6, 11–13], and that lineage-specific expression of cell surface receptors promotes migration along this path [5]. Indeed, several receptor–ligand pairs, including the Slit/ROBO [14], Eph/ephrin [11], endothelin/EDNR [11], and Steel factor/KIT [15, 16] families have been implicated in permitting access to the dorsolateral path. By contrast, there are two competing models to explain the timing at which ventrally migrating NCCs become specified towards the sensory or sympathetic lineages. On the one hand, it is proposed that this population of NCCs are specified prior to or at the time of delamination, and that lineage-specific properties promote migration along restricted pathways. This notion is derived from the findings that neurogenin 2 (Ngn2)-expressing NCCs are strongly biased towards a sensory fate in mice [17], and that nociceptive neurons arise specifically from contralaterally migrating NCCs within the chick neural tube [18]. Specification of pre-migratory NCCs is also supported by lineage-tracing experiments detailing a spatiotemporal fate map of sympathetic- and sensory-fated cells within the chick dorsal neural tube [19]. However, using *in vivo* optical imaging to analyse the behaviour of single cells, McKinney and colleagues recently found that pre-migratory NCCs in the chick lack positional identity or fate prior to delamination [20], but rather, they migrate from the neural tube stochastically and rapidly change their expression profiles as they migrate. This and other lineage/transplantation studies in chick support an alternative model in which migrating NCCs are proposed to be multipotent, becoming specified by their environment only as they migrate towards or at their target regions [21–23].

The molecular cues that cause some ventrally migrating NCCs to remain near the neural tube to form sensory ganglia, while others continue migrating to adopt other fates (e.g. sympathetic ganglia) remain ill defined. In chick, the chemokine receptor CXCR4 is expressed in a specific subset of early migrating NCCs to control their migration towards a source of SDF1 around the anlagen of the sympathetic ganglia [24, 25]. Neuropilins (Nrp1 and Nrp2) are transmembrane receptors for guidance molecules of the class 3 semaphorin (SEMA3) family and for heparin-binding isoforms of vascular endothelial growth factor (VEGF) [26]. Nrp1 and Nrp2 are expressed in both cranial NCCs and ventrally migrating trunk NCCs, and are required for proper segmentation of the peripheral nervous system [27–30]. Notably, phenotypic analysis of single *Nrp1* or *Nrp2* knockout embryos identified distinct migration defects in different NCC lineages. In the head, NCCs emigrating from r4 are misguided in *Nrp1* knockout embryos [31], while NCCs emigrating from r2 are misguided in *Nrp2* knockout embryos [31, 32]. Accordingly, neurons arising from these populations of cranial NCCs are ectopically positioned within neuropilin knockout embryos: neurons from the facio-acoustic ganglia (VII-VIII) are ectopically positioned in *Nrp1* knockout embryos, while neurons from the trigeminal ganglia (V) are ectopically positioned in *Nrp2* knockout embryos [31, 32]. These lineage-specific NCC defects also extend to the trunk, where *Nrp1* knockout embryos have ectopically placed neurons in the sympathetic and sensory nervous systems, and *Nrp2* knockout embryos have misplaced neurons in the sensory nervous system [28, 33, 34]. In combination with expression analyses, these observations suggest that neuropilins may be required for the migration of distinct populations of NCCs, and that this may be controlled by the restricted expression of Nrp1 and Nrp2 in NCCs biased towards sympathetic and/or sensory fates [28]. However, to date, this notion has lacked definitive support from co-expression analyses and fate mapping of neuropilin-expressing NCCs.

Here we have combined *in situ* hybridisation, immunohistochemistry and lineage-tracing analyses to directly compare the expression of Nrp1 and Nrp2 in migrating NCCs. Our results demonstrate that these receptors are expressed in a non-overlapping pattern within different populations of cranial and trunk NCCs. Whereas Nrp1 is expressed in cranial NCCs migrating out of hindbrain r4 and within trunk NCCs migrating towards the sympathetic and sensory ganglia, Nrp2 is preferentially expressed in cranial NCCs migrating out of r2 and in trunk NCCs migrating towards and positioned within the sensory ganglia. To determine whether Nrp2-expressing NCCs are fate-restricted towards the sensory lineage, we generated a tamoxifen-inducible Cre/LoxP tracing system to genetically label and lineage-trace this population of cells. In combination with our detailed expression analyses and previous phenotypic analyses [33, 34], our finding that Nrp2-expressing NCCs give rise to sensory neurons in the DRG and not the sympathetic chain identifies an essential role for neuropilins in coordinating NCC migration with fate specification.

## Results and discussion

### Nrp1 and Nrp2 are expressed in different populations of cranial NCCs

**Figure 1:**
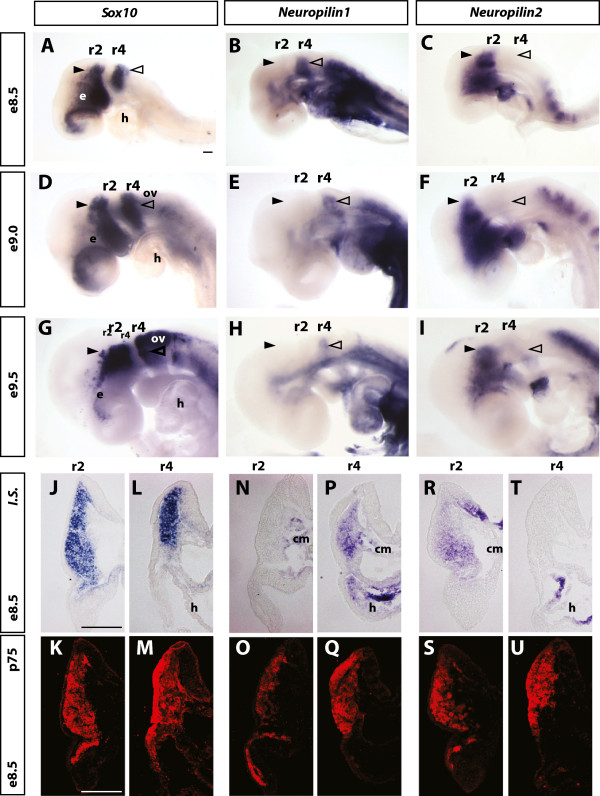
***Nrp1*** **and** ***Nrp2*** **are expressed in different populations of cranial NCCs. (A-I)** Whole mount *in situ* hybridisation of E8.5 to E9.5 wild-type embryos to determine the expression profiles of *Nrp1* and *Nrp2*. **(A, D, G)** *Sox10* labelled all migrating cranial NCCs and defined the r2 (open arrowhead), r4 (closed arrowhead) and r6 populations at E8.5, E9.0, and E9.5 as well as the otic vesicle (ov) at E9.5. **(B, E, H)** *Nrp1* was specifically expressed in the r4 stream of NCCs and not in the r2 stream. **(C, F, I)** *Nrp2* expression was restricted to the r2 stream of NCCs and was lacking in the r4 stream at E8.5 and E9.0. At E9.5, low levels of *Nrp2* could be detected in the tissue ventral to r4. **(J-U)** Transverse *in situ* hybridisation sections of E8.5 embryos through the regions of the r2 and r4 streams counterstained for the neurotrophin receptor p75. **(J-M)** *Sox10* and p75 identified all migrating NCCs. p75 staining was also evident in the epithelia in close association with migrating NCCs in the region ventral to r4. **(N-Q)** Transverse sections confirmed that *Nrp1* is specifically expressed in the r4 NCC stream as well as the developing heart and cranial mesoderm (cm). **(R-U)** Transverse sections confirmed that *Nrp2* was restricted to the r2 stream of NCCs as well as the neural tube, cranial mesoderm, and developing heart. IS, *in situ* hybridisation; e, eye; h, heart, r2, rhombomere 2; r4, rhombomere 4; ov, otic vesicle; e, eye. Scale bar = 100 μm.

Previous studies, including our own, demonstrated that *Nrp1* and *Nrp2* knockout mice have non-overlapping phenotypes in different cranial NCC derivatives [31, 32]. To address if these receptors may be expressed in distinct populations of cranial NCCs, and thereby explain the distinct migration phenotypes of neuropilin knockout embryos, we completed whole mount *in situ* hybridisation on wild-type embryos between embryonic day (E) 8.5 to E9.5. By comparing the expression profiles of *Nrp1* and *Nrp2* with that of the pan-NCC marker *Sox10*, we found that neuropilins are expressed in different populations of cranial NCCs. At E8.5 and E9.0, the time at which NCCs have delaminated from the neural tube and begun to migrate into the branchial arch tissue, the expression of *Nrp1* was restricted to the stream of NCCs emigrating out of r4, while *Nrp2* was expressed reciprocally within NCCs emigrating out of r2 (Figure 1A-F). At E9.5, this restricted expression pattern was maintained; however, low levels of *Nrp2* could also be detected in or around the r4 stream of NCCs (Figure 1G-I). In addition to this presumptive staining within NCCs, *Nrp1* expression was identified in the heart, gut, and arteries (Figure 1B, E, H), while *Nrp2* expression was evident in the dorsal regions of the heart (Figure 1C, F, I).

To investigate whether neuropilins are expressed in NCCs and not other branchial arch tissue such as the surface epithelia or cranial mesoderm, we sectioned E8.5 embryos transversely through the r2 and r4 regions, and counterstained with antibodies specific to the neurotrophin receptor p75. *Sox10* and p75 were co-expressed in all migrating cranial NCCs at this stage of development (Figure 1J-M). Consistent with the results of whole mount staining, *Nrp1* expression was identified specifically in NCCs emigrating out of r4 and also within non-NCC tissues such as the cranial mesoderm and the developing heart (Figure 1N, P). Conversely, *Nrp2* expression was restricted to NCCs emigrating out of r2 and additional tissues such as the cranial mesoderm, neural tube, and bulbus cordis region of the developing heart (Figure 1R, T).

**Figure 2:**
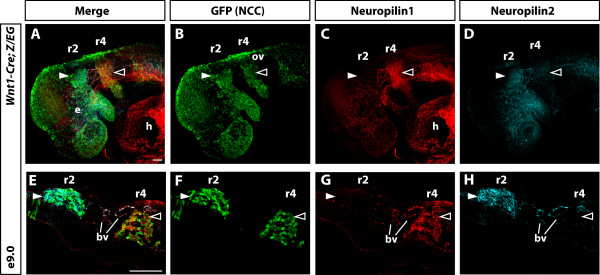
**Nrp1 and Nrp2 expression defines distinct cranial NCC populations along the anteroposterior axis. (A-D)** Whole mount immunofluorescence analysis of Nrp1 and Nrp2 on E9.0 *Wnt1Cre; Z/EG* embryos in which all NCCs were labelled by GFP expression **(B)**. In addition to staining of NCCs, both Nrp1 and Nrp2 were present in blood vessels within and around the r2 and r4 streams. **(C-D)** Antibody staining showed that Nrp1 expression was restricted to the r4 stream of NCCs (closed arrowhead) and Nrp2 was restricted to the r2 stream of NCCs (open arrowhead). **(E-H)** Longitudinal sections through the head confirmed that Nrp1 expression was indeed within NCCs of the r4 stream and not in the r2 stream (delta), while Nrp2 staining was within NCCs of the r2 stream and not in the r4 stream (closed arrowhead). Nrp1 and Nrp2 antibodies also labelled the major blood vessels (bv). e, eye; ov, otic vesicle. Scale bar = 100 μm.

To compare expression of Nrp1 and Nrp2 in NCCs, we immunostained embryos generated by crossing *Wnt1Cre* [35] mice with *Z/EG* [36] reporter mice to specifically label all NCCs and their derivatives with green fluorescent protein (GFP). Whole mount immunofluorescence staining clearly demonstrated that Nrp1 and Nrp2 are expressed in a reciprocal pattern, replicating the mRNA expression seen in our previous *in situ* hybridisation analysis (Figure 2A-D). Immunostaining also identified expression of Nrp1 and Nrp2 in blood vessels around the r2 and r4 migrating NCCs (Figure 2A-D). Longitudinal sections through the head confirmed this restricted expression along the anteroposterior axis, as well as expression within the developing vasculature (Figure 2E-H). The membrane localisation of neuropilins is consistent with their role as cell surface receptors for guidance molecules of the SEMA3 and VEGF families [30]. Taken together, our *in situ* hybridisation, immunohistochemical, and lineage-tracing analyses confirm that neuropilins are expressed in different populations of cranial NCCs that are defined by their position along the anteroposterior axis. These restricted expression profiles therefore explain the disparate phenotypes of neuropilin knockout mice in which *Nrp1* has ectopically placed neurons of the facio-acoustic cranial ganglia (VII-VIII) and *Nrp2* has ectopically placed neurons of the trigeminal ganglia (V) [31, 32]. Future work addressing how the expression of neuropilins is controlled should provide essential clues to the mechanisms dividing cranial NCCs into distinct populations.

### Nrp1 and Nrp2 are expressed in different populations of trunk NCCs

**Figure 3:**
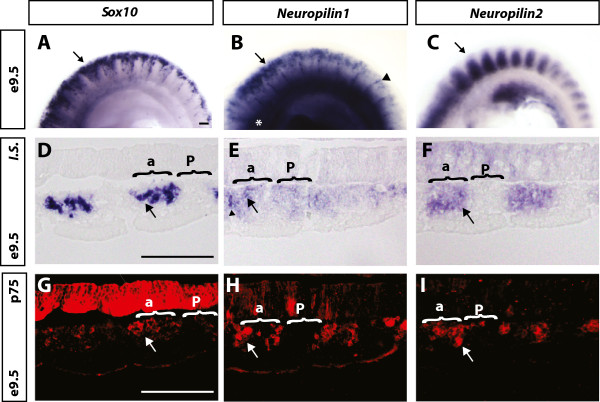
***Nrp1*** **and** ***Nrp2*** **are expressed in trunk NCCs at the same axial level. (A-C)** Lateral view of whole mount *in situ* hybridisation at E9.5 for *Sox10, Nrp1,* and *Nrp2.* **(A)** *Sox10* whole mount *in situ* hybridisation specifically labelled NCCs migrating through the anterior half of the somite (black arrow). **(B)** *Nrp1 in situ* hybridisation labelled NCCs (black arrow), intersomitic blood vessels (white arrowhead) and the dorsal aorta (asterisks). **(C)** *Nrp2* was expressed within the anterior half of the somite (black arrow). **(D-I)** Longitudinal sections of *Nrp1* and *Nrp2 in situ* hybridisation over the hind limb counterstained with antibodies to p75. **(D, G)** p75 expression labelled *Sox10*-positive NCCs in the anterior half of the somite (white and black arrow, respectively). **(E, H)** *Nrp1* had its highest expression in cells that also expressed p75 (black and white arrows), and was also expressed in the somitic tissue and intersomitic blood vessels. (F, I) *Nrp2* was expressed in the anterior half of the somite within NCCs and also in the somitic tissue (black and white arrows). a, anterior; IS, i*n situ* hybridisation; p, posterior. Scale bars = 100 μm.

*Nrp1* and *Nrp2* knockout embryos also displayed disparate phenotypes in different trunk NCC derivatives [33, 34]. To explore if neuropilins are also expressed in different populations of ventrally migrating trunk NCCs, we compared their expression with that of the pan-NCC markers *Sox10* and p75 over the forelimb region of E9.5 embryos. *In situ* hybridisation confirmed that *Nrp1* was expressed within NCCs migrating in the anterior half of the somite and in non-NCC derivatives such as the dorsal aorta and intersomitic blood vessels (Figure 3B). *Nrp2* was robustly expressed in the anterior half of the somite in a pattern that partially overlapped with *Sox10* (Figure 3C). To determine if neuropilins are expressed within NCCs or the supporting somitic tissue, we next counterstained longitudinal *in situ* hybridisation sections with anti-p75 antibodies (Figure 3D-I). In addition to labelling *Sox10*-expressing NCCs, p75 also recognised neuroepithelial cells within the neural tube (Figure 3D, G). Co-staining of *Nrp1* and p75 within the anterior half of the somite indicated that *Nrp1* was expressed in trunk NCCs migrating along the ventral path (Figure 3E, H). In addition, *Nrp1* was also expressed in the posterior somite and within intersomitic blood vessels. *Nrp2* was broadly expressed throughout the entire anterior half of the somite, with some cells co-staining with p75 (Figure 3F, I).

**Figure 4:**
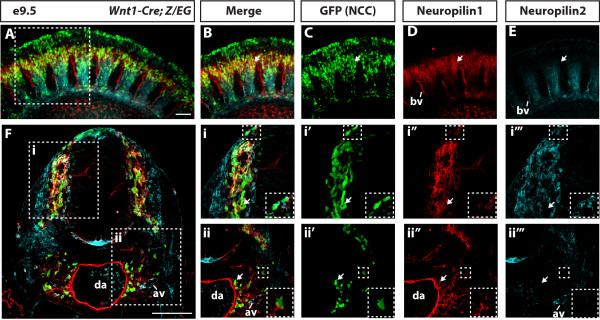
**Nrp1 and Nrp2 are expressed in distinct populations of trunk NCCs. (A-E)** Lateral view of whole mount immunofluorescence staining of Nrp1, Nrp2 and trunk NCCs at E9.5. **(C)** GFP labelled all NCCs migrating in the anterior half of the somite. **(D)** Nrp1 was expressed in many GFP-positive NCCs and intersomitic vessels. **(E)** Nrp2 was expressed in the anterior half of the somite and within NCCs. Notably, Nrp1 had high expression in NCCs that had either low or negative Nrp2 expression (white arrow). **(F)** Transverse sections through the anterior half of the somite over the forelimb region showed that neuropilins were expressed within different populations of NCCs. NCCs migrating through the somite in and around the anlage of the dorsal root ganglia (Fi-i′′′) consisted of cells with high Nrp2 and no Nrp1 expression (inset) and conversely, with high levels of Nrp1 and no expression of Nrp2 (white arrow). (Fii-ii′′′) NCCs migrating towards the dorsal aorta (inset) or condensing in the region of the sympathetic ganglia (white arrow) preferentially expressed Nrp1. av, anterior cardinal vein; bv, blood vessel; da, dorsal aorta. Scale bars = 100 μm.

To investigate whether neuropilins are co-expressed or differentially expressed within distinct populations of ventrally migrating NCCs, we performed immunofluorescence analysis on E9.5 *Wnt1Cre; Z/EG* embryos. Whole mount staining for GFP, Nrp1 and Nrp2 identified mixed populations of NCCs expressing the neuropilin receptors (Figure 4A-E). Consistent with our *in situ* hybridisation analysis results, Nrp1 was identified in NCCs in the anterior half of the somite, the intersomitic blood vessels, and the dorsal aorta (Figure 4D). Nrp2 was also expressed in NCCs in the anterior half of the somite in addition to the intersomitic blood vessels. Notably, there was limited overlap of the Nrp1 and Nrp2 expression domains, and NCCs expressing high levels of Nrp1 with low or absent Nrp2 could be identified in the dorsal regions of the somite (Figure 4A-E arrow).

To define the expression of neuropilins within individual trunk NCCs, we next analysed transverse sections of E9.5 embryos taken from the anterior half of the somite at the level of the developing forelimb (somites 13–14, Figure 4F). Consistent with the whole mount staining results, we identified Nrp1 within migrating NCCs, the dorsal aorta, and the periaortic mesenchyme. Nrp2 was also identified in NCCs and NCC precursors within the neural tube, the floor plate, somitic mesenchyme, and anterior cardinal veins. Notably, NCCs located around the dorsal aorta that had sympathetic fate preferentially expressed Nrp1 (Figure 4Fii, white arrow). Moreover, NCCs with presumptive sympathetic fate migrating towards the dorsal aorta also preferentially expressed high levels of Nrp1 (Figure 4Fii and inset). By contrast, NCCs migrating within the somite and/or arresting within the anlagen of the DRG expressed various combinations of the neuropilin receptors. Although the majority of the NCCs within the area of the DRG co-expressed Nrp1 and Nrp2, we also identified NCCs within and around the DRG that preferentially expressed either Nrp1 (Figure 4Fi, arrow) or Nrp2 alone (Figure 4Fi and inset). NCCs expressing only Nrp1 were mostly located at the ventral side of the DRG in close association with the neural tube, while NCCs expressing only Nrp2 were located at the prospective dorsal root entry zone.

**Figure 5:**
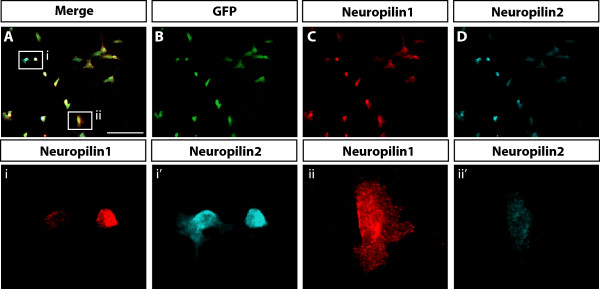
**NCCs express combinations of Nrp1 and Nrp2. (A-D)** FACS of GFP-positive trunk NCCs from E9.5 *Wnt1Cre; Z/EG* embryos plated on fibronectin and stained with antibodies to Nrp1 and Nrp2. **(B)** GFP was identified in all FACS-sorted cells. **(C-D)** Nrp1 and Nrp2 were co-expressed in most NCCs. (i-i′) Some NCCs expressed high levels of Nrp2 and low levels of Nrp1. (ii-ii′) Some NCCs expressed high levels of Nrp1 and low levels of Nrp2. Scale bar = 100 μm.

We tested the notion that NCCs express combinations of the neuropilin receptors by purifying GFP-positive trunk NCCs from E9.5 *Wnt1Cre; Z/EG* embryos, using fluorescence-activated cell sorting (FACS). Consistent with our *in vivo* expression analysis, counterstaining of Nrp1 and Nrp2 identified three populations of trunk NCCs based on their neuropilin expression profiles: 1) Nrp1 high/Nrp2 high, 2) Nrp1 high/Nrp2 low, and 3) Nrp2 high/Nrp1 low (Figure 5). Given the different locations of these cells within the embryo, our expression profiling also suggests that each of these NCC types may give rise to different derivatives. We therefore propose that the majority of Nrp1 high/Nrp2 high NCCs form neurons and glia of the DRG, Nrp2 high/Nrp1 low NCCs form boundary cap cells at the dorsal root entry zone, and Nrp1 high/Nrp2 low NCCs form neurons and glia of the sympathetic nervous system.

Upon reaching the dorsal aorta, NCCs commence differentiation into *bona fide* sympathetic neurons via the elevated expression of the basic helix-loop-helix transcription factor MASH1. NCC derivatives that have initiated this differentiation program also begin to express Nrp2 [37]. Consistent with this developmental progression, our expression analysis in sections from more developmentally advanced regions of E9.5 embryos (somites 5–6 that are anterior to the forelimb) identified a small number of GFP-positive cells at the dorsal aorta co-expressing Nrp1 and Nrp2 (not shown).

Previous phenotypic analysis of neuropilin knockout embryos found that Nrp1 is required for correct positioning of both sensory and sympathetic neurons, while Nrp2 is required for positioning of sensory neurons [28, 33, 34]. Our expression analysis therefore explains the disparate phenotypes between the two types of knockout mice, and further suggests that neuropilins may play essential roles in coordinating NCC migration with fate specification.

### Generation of an inducible lineage-tracing Nrp2 transgenic mouse model

**Figure 6:**
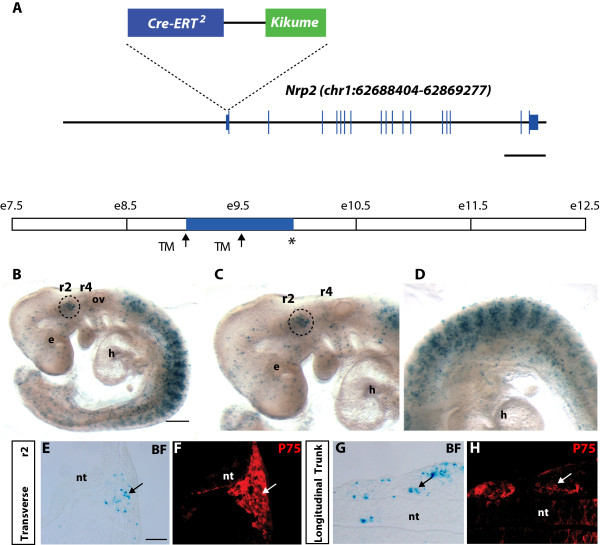
**Generation of Nrp2-CreERT2/Kikume lineage-tracing mice. (A)** The *Nrp2- CreERT2/Kikume* transgenic construct consisted of a BAC (RP24-250G22) encompassing 62 kb upstream of the *Nrp2* start codon and 4 kb downsteam of the 3′ UTR. A cassette consisting of the sequences for *CreERT2*, *IRES ,*and *Kikume* tagged in-frame to the FLAG tag was inserted in place of the start codon of *Nrp2*. **(B-D)** Whole mount X-gal staining of E10.0 *Nrp2-CreERT2/Kikume* X *R26R* embryos injected with tamoxifen (TM) at E9.0 and E9.5 (arrows). **(B,C)** In the head, X-gal staining was present in NCCs condensing into the anlagen of the Vth cranial ganglia (dashed circle) and not the VII-VIIIth cranial ganglia. **(B, D)** In the trunk, X-gal staining was present within the anterior somite. **(E-H)** Whole mount embryos were sectioned and counterstained with p75. **(E-F)** Transverse sections through r2 showed that LacZ expression occurred within NCCs (arrow). **(G-H)** Longitudinal sections through the trunk show that LacZ expression occurred within migrating trunk NCCs (arrow). e, eye; h, heart; nt, neural tube; ov, otic vesicle; *, collection of embryos. Scale bars = A, 10 kb; B, 500 μm; D, 100 μm.

To identify the fate of neuropilin-expressing NCCs and to determine if Nrp2-expressing trunk NCCs are biased towards the sensory neuronal lineage, we generated a tamoxifen-inducible Nrp2 lineage-tracing mouse model. A *Nrp2-CreERT2/Kikume* transgenic construct was generated by replacing the ATG start codon of the *Nrp2* gene with the entire *CreERT2 IRES Kikume* sequence followed by a polyadenylation signal sequence, thus placing *CreERT2 IRES Kikume* under control of the endogenous regulatory elements contained in the *Nrp2* gene locus (Figure 6A). The engineered construct was used to generate a transgenic founder mouse that was identified by PCR using primers specific for *CreERT2*. The offspring from the founder line appeared grossly normal, and the transgene was transmitted as predicted by Mendelian ratios.

To permanently label and follow the fate of *Nrp2* expressing cells throughout embryogenesis, *Nrp2-CreERT2/Kikume* transgenic mice were crossed with *R26R LacZ* reporter mice to create *Nrp2-CreERT2/Kikume; R26R LacZ* embryos. Upon addition of tamoxifen to these embryos, CreERT2 translocated to the nucleus to promote stable expression of β-galactosidase (lacZ) in cells and descendants of cells endogenously expressing *Nrp2* at the time of injection. No lacZ labelling was detected without tamoxifen injection (data not shown).

We next determined that intraperitoneal injection of tamoxifen at 2–4 mg per 40 g body weight to pregnant dams was sufficient to label Nrp2-expressing cells without resulting in embryonic lethality. As expected, higher doses of tamoxifen injected at E7.5 to E8.5 had detrimental effects on embryos.

Having optimised the dosage of tamoxifen, we first validated that the transgene recapitulated the Nrp2 expression profile in NCCs. Tamoxifen was injected at E9.0 and E9.5, and embryos collected at E10.0. Analysis of X-gal staining in whole mount embryos demonstrated that the transgene is expressed in a highly overlapping pattern to endogenous Nrp2 (n = 12; compare Figure 6B with Figures 1I, 2D, 3C, and 4E). Indeed, the transgene expression pattern replicated the expression of Nrp2 within a distinct striped pattern in the trunk and within NCCs migrating out of r2 (Figure 6B-D). To confirm that the expression was occurring within NCCs, we sectioned E10.0 embryos and counterstained with the NCC marker p75 (Figure 6E-H). Transverse sections through r2 and longitudinal sections through the trunk confirmed transgene expression within migrating NCCs, somitic tissue, and other cell types within the epidermis (detailed analysis of transgene expression in other tissues will be presented elsewhere). These findings demonstrate that the regulatory elements controlling expression of Nrp2 within NCCs are present within the bacterial artificial chromosome (BAC) used to make this transgenic animal. This mouse model provides an ideal resource for lineage-tracing studies and for the temporal removal of floxed genes in specific NCC populations and other cell types in which Nrp2 is expressed.

### Nrp2-expressing NCCs give rise to neurons and glia in the trigeminal ganglia

**Figure 7:**
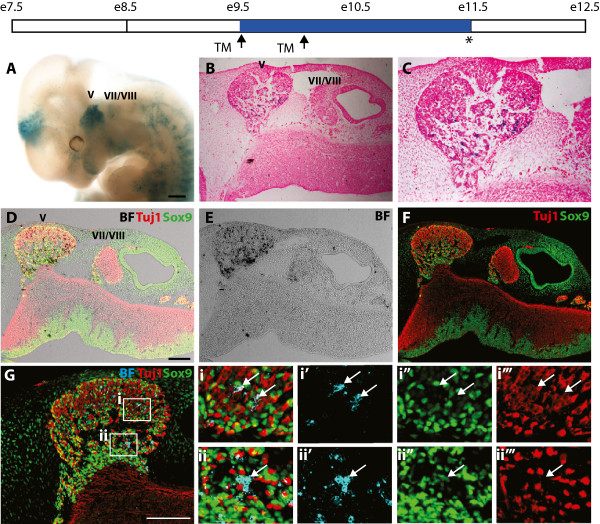
**Nrp2-expressing NCCs give rise to neurons and glia of the trigeminal ganglia. (A)** Whole mount X-gal staining of E11.5 *Nrp2-CreERT2/Kikume* X *R26R* embryos injected with tamoxifen (TM) at E9.5 and E10.0 (arrows). Staining was restricted to the trigeminal ganglia (V) that were derived from the r2 stream of NCCs. Notably, staining was absent from derivatives of the r4 stream of NCCs such as the facio-acoustic ganglia (VII-VIII). **(B-C)** Longitudinal sections through the head of *Nrp2-CreERT2/Kikume* X *R26R* embryos counterstained for X-gal and eosin confirmed that transgene expression was restricted to the trigeminal ganglia (V) and lacking in the facio-acoustic ganglia (VII-VIII). **(D-G)** Serial sections to that in **(B)** counterstained for X-gal, Tuj1, and Sox9 confirmed transgene expression within neurons and glia of the trigeminal ganglia (V) and not within the facio-acoustic ganglia (VII-VIII). **(D)** Bright-field (BF) images of X-gal staining overlaid with Tuj1 and Sox9. **(G)** BF images were colour-inverted to cyan to promote colocalisation analysis. (Gi-Gi′′′ X-gal-positive descendants of Nrp2-expressing NCCs co-expressed Tuj1 (arrow). (Gii-Gii′′′) X-gal-positive descendants of Nrp2-expressing NCCs co-expressed Sox9 (arrow). Scale bars = A, 500 μm; D, G, 100 μm.

Using this inducible lineage-tracing mouse model, we first determined the derivatives of Nrp2-expressing NCCs in the head. Tamoxifen was administered to pregnant dams when embryos were at E9.5 and E10.0, and later collected at E11.5. Consistent with Nrp2 being restricted to specific cranial NCC subpopulations, analysis of whole mount embryos revealed distinct X-gal staining in the trigeminal (V) ganglia that are derived from NCCs migrating out of r2 (n = 8, Figure 7A). By contrast, minimal staining was detected in r4 NCC derived structures such as the facio-acoustic ganglia (VII-VIII). X-gal staining also identified additional Nrp2-expressing cells in the forebrain region. Longitudinal sections counterstained with eosin confirmed the restriction of Nrp2-expressing NCCs to the trigeminal ganglia (V) (Figure 7B-C).

To define the fate of Nrp2-expressing NCCs, we next counterstained longitudinal X-gal-stained sections with antibodies specific for neurons (anti-Tuj1 antibodies) and glia (anti-Sox9 antibodies) (Figure 7D-G). Under high magnification, Nrp2-expressing NCCs were found to give rise to both Tuj1-positive neurons and Sox9-positive glia within the trigeminal ganglia (V) (Figure 7Gi, Gii, respectively). Taken together with our expression profiling results, we conclude that Nrp2 expression is restricted to NCCs emigrating out of hindbrain r2. As we were unable to detect X-gal staining in other derivatives of the r2 stream such as bone and cartilage of the mandible, our results suggest that Nrp2-expressing NCCs preferentially give rise to neuroglial lineages. In addition, both whole mount staining and staining of transverse sections (not shown), suggest that Nrp2-expressing NCCs give rise to Schwann cells along the axonal tracts of the trigeminal ganglia (V).

### Nrp2-expressing trunk NCCs are biased towards sensory ganglia

**Figure 8:**
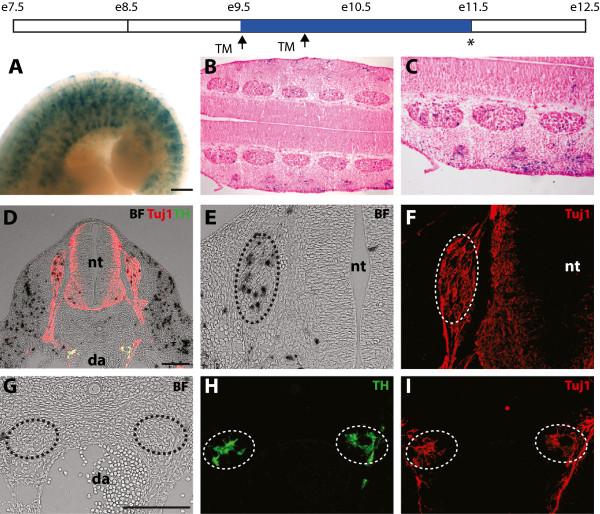
**Nrp2-expressing NCCs give rise to neurons and glia of the dorsal root ganglia. (A)** Whole mount X-gal staining of E11.5 *Nrp2-CreERT2/Kikume* X *R26R* embryos injected with tamoxifen (TM) at E9.5 and E10.0 (arrows). Staining was identified in presumptive skeletal muscle, skin, and DRG. **(B-C)** Longitudinal sections anterior to the hind limbs counterstained with eosin confirmed X-gal staining within sensory neurons and glia of the DRG, skeletal muscle and skin. **(D-I)** Transverse sections anterior to the hind limbs counterstained with Tuj1 and TH demonstrated X-gal staining within sensory neurons of the DRG (**E-F**, dashed circle) but not within sympathetic neurons (**G-I**, dashed circle). da, dorsal aorta; nt, neural tube. Scale bars = A, 500 μm; D & G, 100 μm.

Our expression analysis raised the hypothesis that ventrally migrating trunk NCCs expressing Nrp2 are biased towards neurons and glia of the sensory ganglia. To test this notion, we completed fate-mapping studies with the Nrp2-inducible lineage-tracing mouse model. Tamoxifen was administered to pregnant dams when embryos were E9.5 and E10.0, and these were later collected at E11.5, a stage at which the sensory and sympathetic ganglia have started to condense. Consistent with the staining of Nrp2 in NCCs and paraxial mesoderm at E9.5, X-gal staining identified derivatives of Nrp2-expressing cells within the DRG and skeletal muscle (Figure 8A). Eosin staining of longitudinal sections anterior to the hind limb clearly demonstrated X-gal staining within the DRG (Figure 8B-C). Transverse sections through the same axial level were also counterstained with the pan neuronal marker Tuj1 and the sympathetic specific neuronal marker tyrosine hydroxylase (TH) (Figure 8D). In all cases examined (n = 3), X-gal staining was robustly observed in the DRG (Figure 8D-F). By contrast, we were unable to observe X-gal staining in sympathetic ganglia (Figure 8G-I). This finding demonstrates that Nrp2-expressing NCCs are strongly biased towards neurons and glia of the DRG.

Our fate-mapping results of Nrp2-expressing NCCs are strikingly similar to that presented for lineage tracing of Ngn2-expressing NCCs in mice [17]. In the latter, Ngn2-positive cells were also biased towards sensory ganglia. As Ngn2 is a basic helix-loop-helix transcription factor that plays an essential role in sensory neuronal differentiation [38], it will be of interest to determine if Nrp2 is also involved in the same molecular pathway within NCCs.

## Conclusions

**Figure 9:**
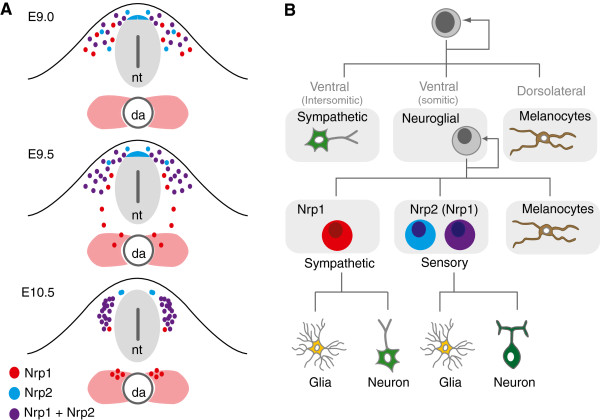
**Neuropilins define molecularly distinct populations of ventrally migrating trunk NCCs. (A)** Schematic detailing the neuropilin expression profiles in migrating trunk NCCs. NCCs migrating to the dorsal aorta (da) give rise to the sympathetic ganglia, while NCCs that stall within the somite alongside the neural tube (nt) give rise to sensory neurons of the DRG. Trunk NCCs at the level of the forelimb begin to delaminate from the neural tube at E8.5, and by E9.0, have started to migrate within the somite. At E9.5, some NCCs have already reached the dorsal aorta, while others have stalled in the anlagen of the DRG. At E10.5, NCCs with sympathetic fate have condensed, while NCCs with sensory fate have started differentiating in the DRG. Expression profiling identified distinct populations of NCCs with Nrp1 (red), Nrp2 (blue), and Nrp1/Nrp2 (purple). Nrp1-expressing cells preferentially migrate towards the dorsal aorta while Nrp2-expressing cells stall within the area of the DRG. Nrp2 was also expressed in presumptive NCC precursors within the dorsal neural tube. **(B)** Schematic diagram detailing the fate restriction of trunk NCCs. NCCs delaminate from the neural tube to migrate along two separate paths. Ventrally migrating NCCs initially travel through the intersomitic space to seed the sympathetic ganglia, and then switch to travel ventrally through the anterior half of the somite to give rise to sensory ganglia, sympathetic ganglia, and melanocytes. NCCs migrating dorsolaterally also give rise to melanocytes. Expression, fate-mapping, and phenotypic studies suggest that Nrp2 (Nrp2 alone (blue) and Nrp1/Nrp2 (purple)) is a marker of NCCs biased towards sensory ganglia. In addition, expression and phenotypic studies suggest that Nrp1 (red) is a marker of ventrally migrating NCCs that give rise to sympathetic ganglia.

The work presented here identified molecularly distinct populations of cranial and trunk NCCs based on their expression profiles of the cell surface receptors Nrp1 and Nrp2. In combination with the recent analyses of Nrp1 and Nrp2 knockout mice [31–34], this work further identifies essential roles for neuropilins in sorting specific populations of NCCs to their final destinations within the embryo (Figure 9A). In the head, Nrp1 is expressed in NCCs emigrating from r4, and is required for the migration and condensation of neurons and glia of the facio-acoustic ganglion (VII-VIII) [31]. By contrast, Nrp2 is expressed in NCCs emigrating from r2, and is required for the migration and condensation of neurons and glia of the trigeminal ganglion (V) [32].

In addition to their differential expression in restricted populations of NCCs along the anteroposterior axis, neuropilins also label divergent populations of NCCs along the dorsoventral axis (Figure 9A). Trunk NCCs migrating along the ventral path through the anterior somite consist of a mixed population of sympathetic and sensory progenitors. Our finding that Nrp1 and Nrp2 expression correlates with sympathetic and sensory progenitors, and that Nrp2-expressing NCCs are biased towards neurons and glia of the sensory nervous system therefore suggests that neuropilins, possibly in combination with other guidance molecules such as CXCR4 [25], provide part of the molecular machinery to guide ventrally migrating NCC precursors to correct target regions of the embryo (Figure 9B). Indeed, phenotypic analysis of neuropilin knockout mice is in strong agreement with this notion [33, 34].

Our study identified three distinct NCC sub-types within and around the DRG that can be segregated based on their neuropilin expression profiles. While Nrp2-expressing NCCs (Nrp2-high/Nrp1-high and Nrp2-high/Nrp1-low) were traced to the DRG it will be important to complete the inverse experiment with lineage tracing of Nrp1-expressing NCCs to determine if this population is also fate-restricted.

Our expression analysis also identified Nrp2 within presumptive NCC precursors in the dorsal neural tube (Figure 4F). Given that Nrp2 knockout embryos have defects restricted to sensory NCC derivatives, and that Nrp2-expressing NCCs are fate-restricted towards a sensory phenotype, our data could also be taken to support the notion that at least some pre-migratory NCCs are fate-biased prior to delamination. However, as clonal analysis or transplantation of Nrp2-expressing pre-migratory/migrating NCCs into ectopic environments has not yet been achieved, our studies are currently unable to decipher between the two models of when ventrally migrating NCCs are segregated into specified lineages. For example, although the Nrp2-expressing cells may be biased towards a sensory phenotype, they may only commit to this lineage upon reaching their final resting place within the sclerotome.

Taken together with previous reports, our studies demonstrate that neuropilins provide part of the molecular machinery to sort heterogeneous populations of ventrally migrating NCCs towards correct target regions.

## Methods

### Mice

All experimentation was approved by and conducted in accordance with the guidelines of the Animal Ethics Committee of the SA Pathology/Central Adelaide Local Health Network, and followed the Australian code of practice for the care and use of animals for scientific purposes. To obtain embryos of defined gestational ages, animals were mated in the evening, and the morning of vaginal plug formation was counted as embryonic day (E) 0.5. To lineage-trace NCCs and their derivatives throughout development, we crossed *Wnt1Cre* [35] with *Z/EG* [36] mice.

### Generation of Nrp2-CreERT2/Kikume transgenic mice

The BAC clone RP24-250G22, containing 62 kb of DNA upstream of the first exon to 4 kb past the final coding exon of the *Nrp2* locus, was obtained from the BAC resource at Children’s Hospital Oakland Research Institute (Oakland, California, USA). A *CreERT2 IRES FLAG-Kikume* cassette was inserted at the initiation codon of the *Nrp2* coding sequence, which is located within exon 1, by homologous recombination using a targeting vector designed for BAC recombineering [39, 40]. A 5′ homology arm was amplified by PCR with a forward primer 5′-taactagtctcgagctctgggaacacagagctgag-3′ and reverse primer 5′-taactagtagagagcgatccgattacg-3′ and inserted upstream of the *CreERT2 IRES FLAG-Kikume* sequence in plasmid pBS-CreERT2 IRES FLAG-Kikume (Schwarz laboratory). The 5′ homology arm and *CreERT2 IRES FLAG-Kikume* fragment was then sub-cloned to the recombineering vector PL451 using *Xho*I restriction sites. A 3′ homology arm was amplified by PCR with a forward primer 5′-taggatccgtaagcccttcaaagtttttc-3′ and reverse primer 5′-tagcggccgcaaagaatccacacatgtgaaaag-3′, and inserted downstream of *CreERT2 IRES FLAG-Kikume Neo/Kan* in the PL451 vector using unique *Bam*HI/*Not*I sites. This created the *Nrp2- CreERT2/Kikume* PL451 recombineering vector.

The RP24-250G22 BAC was transformed into the recombinogenic *Escherichia coli* strain EL250 and maintained with chloramphenicol resistance. EL250 cells carrying RP24-250G22 BAC were electroporated with *Nrp2-CreERT2/Kikume* PL451, and homologous recombined positive clones were selected with kanamycin and chloramphenicol. The Neo/Kan cassette was removed by L-arabinose induction of flp recombinase, resulting in a *Nrp2-CreERT2/Kikume* BAC. Homologous recombination was confirmed by PCR and sequencing of the BAC using primers that flanked upstream of the *Nrp2* 5′ homology arm and downstream of the 3′ homology arm. High-quality BAC DNA was prepared using the Large Construct Kit (Qiagen, Chadstone, VIC, Australia) and analysed by pulsed field gel electrophoresis, restriction analysis, and BAC end sequencing. Prior to microinjection, the DNA elution buffer was replaced with fresh microinjection buffer (10 mM Tris–HCl, pH 8.0 0.25 mM EDTA, pH 8.0) by microdialysis. The *Nrp2-CreERT2/Kikume* BAC was microinjected into fertilized embryos by standard pronuclear injection techniques at the Transgenic Animal Services Queensland (TASQ, Brisbane, GLD, Australia). Genomic DNA was isolated from tail samples [41], and founder mice carrying the BAC transgene were identified by PCR with primer pairs recognising *CreERT2*: iCre forward 5′-gagagatggatctctgtgtc-3′ and iCre reverse 5′-gacttcatcagaggtggcatc-3′, yielding a 580 bp product. Founders and offspring were subsequently genotyped with the same primer pair. One founder line was produced that transmitted the transgene in a normal mendelian inheritance pattern, with all offspring appearing grossly normal.

### *In situ*hybridisation

Whole mount and section *in situ* hybridisation was performed as described previously [42]. Riboprobes were transcribed from plasmids containing the cDNA sequence for *Sox10*, *Nrp1*, and *Nrp2* [33, 34].

### Immunolabelling

Embryos were fixed in 4% paraformaldehyde in PBS. All sections were cut at a thickness of 14 μm on a CM1850 cryostat (Leica, North Ryde, NSW, Australia) and air-dried for 60 min before staining. For immunolabelling, cryosections or whole mount embryos were blocked in PBS containing 0.2% BSA and 0.5% Triton X-100, and stained with the indicated primary antibodies: rabbit anti-Sox9 1:1000; rabbit tyrosine hydoxylase 1:300 (both Millipore, Batswater, VIC, Australia); goat anti-Nrp1 1:500; rabbit anti-Nrp2 1:500 (both R&D, Minneapolis, MN, USA); rabbit anti-p75-NTR 1:200 (Epitomics, Burlingame, CA, USA); mouse anti β-tubulin isotype III (Sigma-Aldrich, Sydney, NSW, Australia) 1:750; and chicken anti-GFP 1:1000 (Abcam, Melbourne, VIC, Australia). Cyrosections were mounted in Prolong Gold Antifade Reagent containing DAPI (Molecular Probes, Mulgrave, VIC, Australia). Confocal images were acquired under a confocal microscope (LSM 700; Zeiss, Jena, Germany). All images were prepared with Photoshop software (Adobe, San Jose, CA, USA).

### β-Galactosidase staining

Embryos were fixed in PBS containing 4% paraformaldehyde. Whole embryos or cryosections were incubated in staining solution: 19 mM sodium dihydrogen phosphate, 81 mM disodium hydrogen phosphate, 2 mM MgCl_2_, 5 mM EGTA, 0.01% sodium deoxycholate, 0.02% NP-40, 5 mM potassium ferricyanide, 5 mM potassium ferrocyanide, and 1 mg/ml X-gal substrate, at 37°C until blue staining was sufficient. Sections were counterstained with eosin.

### Tamoxifen

Tamoxifen 1 g (Sigma-Aldrich, Sydney, NSW, Australia) was suspended in 5 ml of ethanol, and then dissolved in 45 ml of sunflower oil to produce a final stock at 20 mg/ml concentration. To dissolve the tamoxifen completely, the stock was sonicated in an ice bath for 5 min (with 30 second intervals). Pregnant dams were injected intraperitoneally with 2 mg tamoxifen per 40 g body weight at the indicated time points.

### FACS sorting of primary neural crest cells

Primary NCCs were isolated from E9.5 *Wnt1Cre; Z/EG* embryos as previously described [43]. Trunk regions posterior to somite 10 were dissociated using Tryple Express (Invitrogen, Mulgrave, VIC, Australia) for 10 minutes at room temperature. Dissociated cells were washed twice with Dulbecco’s modified Eagle’s media (DMEM) containing 10% fetal calf serum (FCS), and resuspended in DMEM with 1% FCS for cell sorting. Cell sorting was performed on a Beckman Coulter Epics Altra HyperSort using Expo MultiComp Software (v1.2B; Beckman Coulter, Lane Cove, NSW, Australia) equipped with an Innova 300C water-cooled 488 nm argon laser at 100 mW. Sorting was conducted at room temperature, with the instrument pressurised to 12 psi and equipped with a 100 μm nozzle. Linear forward scatter (FSC) peak signal (pk), width (time of flight (TOF)) and internal signal area (INT) signals were collected to allow for standard scatter and doublet discrimination. Linear side scatter (SSC) and INT signal were collected with a 488/10 band pass filter in the photomultiplier tube (PMT1). Log GFP signal was collected in PMT2 with a 525/25 band pass filter behind a 488 nm long pass dichroic mirror. A gate was drawn on FSC versus SSC plot to exclude debris and dead cells, as discriminated by scatter properties alone. Following this, an FSC pk versus FSC INT plot was examined to allow distinction of single cells. Linearly related cells were gated for further analysis on a GFP versus SSC plot. Cells were collected into DMEM with 10% FCS. After collection, primary NCCs were centrifuged, resuspended in NCC growth media (DMEM F12, 5% chicken embryo extract, 10 mM HEPES, 2% B27, 1% N2, 20 ng/ml insulin-like growth factor (IGF, R&D, Minneapolis, MN, USA), and 100 U/ml Penicillin/Streptomycin (Invitrogen, Mulgrave, VIC, Australia). Cells were plated in this media in Ibidi μ-slide eight-well dishes coated with 50 mg/ml fibronectin (Roche, Basel, Switzerland) at a concentration of 5 × 10^4^ cells/well. After 4 hours, cells were fixed with 4% PFA for 10 minutes, and immunolabelled with anti-Nrp1 and anti-Nrp2 antibodies. Images were acquired on an Olympus IX81 microscope using Cell X-cellence software (Olympus, Edwardstown, SA, Australia).

## Abbreviations

BAC: bacterial artificial chromosome
DRG: Dorsal root ganglia
GFP: Green fluorescent protein
NCC: Neural crest cell
Nrp1: Neuropilin 1
Nrp2: Neuropilin 2
TH: Tyrosine hydroxylase.
